# *In loco* nursing practice inspection costs in a Brazilian setting

**DOI:** 10.1590/1980-220X-REEUSP-2021-0382

**Published:** 2022-01-28

**Authors:** Vera Lucia de Souza Alves, Antônio Fernandes Costa Lima

**Affiliations:** 1 Universidade de São Paulo, Escola de Enfermagem, Programa de Pós-graduação em Gerenciamento em Enfermagem, São Paulo, SP, Brazil.; 2 Universidade de São Paulo, Escola de Enfermagem, Departamento de Orientação Profissional, São Paulo, SP, Brazil.

**Keywords:** Professional Review Organizations, Health Care Coordination and Monitoring, Administrative Claims, Healthcare, Nursing Services, Nursing Staff, Costs and Cost Analysis, Organizaciones de Normalización Profesional, Regulación y Fiscalización en Salud, Reclamos Administrativos en el Cuidado de la Salud, Servicios de Enfermería, Personal de Enfermería, Costos y Análisis de Costo, Organizações de Normalização Profissional, Regulação e Fiscalização em Saúde, Processos Administrativos dos Serviços de Saúde, Serviços de Enfermagem, Recursos Humanos de Enfermagem, Custos e Análise de Custo

## Abstract

**Objective:**

To identify the average direct cost related to the direct labor of the inspectors involved in the “*in loco* inspection” step of the inspection process carried out at the Headquarters of the Regional Nursing Council of São Paulo.

**Method:**

Quantitative, exploratory-descriptive research, in the form of a single case study. The non-probabilistic convenience sample consisted of records of initial and return “*in loco* inspections”, carried out by inspectors working at the Headquarters of The Regional Nursing Council of São Paulo, from January 13, 2020 to March 13, 2020.

**Results:**

The average direct cost of initial *in loco* inspection (N = 182) corresponded to BRL 331.67 (SD = 140.32), ranging from BRL 115.80 to BRL 1071.15, and that of return *in loco* inspection (N = 98) to BRL 256.16 (SD = 130.90), ranging from BRL 77.20 to BRL 694.80. Time and cost variables analysis of initial and return *in loco* inspections showed an alpha significance level of 0.05, and it was possible to statistically state that the time (p ≤ 0.001) and the cost of initial *in loco* inspection (p ≤ 0.001) are higher than those for return *in loco* inspection.

**Conclusion:**

the cost of the step of “*in loco* inspection” will support the Nursing Council in the decision-making process aiming at allocating efficiency of human resources required in the inspection process.

## RESUMO

**Objetivo:**

Identificar o custo direto médio relativo à mão de obra direta dos fiscais envolvidos na etapa “inspeção *in loco*” do processo de fiscalização realizado na Unidade Sede do Conselho Regional de Enfermagem de São Paulo.

**Método:**

Pesquisa quantitativa, exploratório-descritiva, na modalidade de estudo de caso único. A amostra de conveniência, não probabilística, foi constituída por registros de “inspeções *in loco”*, iniciais e de retorno, realizadas por fiscais atuantes na Unidade Sede, no período de 13/01/2020 a 13/03/2020.

**Resultados:**

O custo direto médio da inspeção *in loco* inicial (N = 182) correspondeu a R$ 331,67 (DP = 140,32), variando de R$ 115,80 a R$ 1071,15, e da inspeção *in loco* de retorno (N = 98) a R$ 256,16 (DP = 130,90), variando entre R$77,20 e R$ 694,80. A análise das variáveis tempo e custo das inspeções *in loco* iniciais e de retorno evidenciou nível de significância alfa de 0,05, sendo possível afirmar estatisticamente que o tempo (p ≤ 0,001) e o custo da inspeção *in loco* inicial (p ≤ 0,001) são maiores do que os da inspeção *in loco* de retorno.

**Conclusão:**

o custeio da etapa “inspeção *in loco*” subsidiará o Conselho no processo decisório visando à eficiência alocativa dos recursos humanos requeridos no processo de fiscalização.

## RESUMEN

**Objetivo:**

Identificar el coste medio directo relacionado con la labor directa de los inspectores involucrados en la etapa de “inspección in loco” del proceso de inspección realizado en la Unidad Sede del Consejo Regional de Enfermería de São Paulo.

**Método:**

Investigación cuantitativa, exploratoria-descriptiva, en forma de estudio de caso único. La muestra de conveniencia no probabilística consistió en registros de “inspecciones in loco”, iniciales y de retorno, realizadas por inspectores que trabajan en la Sede, desde el 13/01/2020 al 13/03/2020.

**Resultados:**

El coste medio directo de la inspección inicial (N = 182) correspondió a R$ 331,67 (DP = 140,32), con un rango de R$ 115,80 a R$ 1071,15, y el de la inspección de retorno (N = 98) a R$ 256,16 (DP = 130,90), oscilando entre R$ 77,20 y R$ 694,80. El análisis de las variables tiempo y coste de las inspecciones inicial y de retorno indicó un nivel de significancia alfa de 0.05, y fue posible afirmar estadísticamente que el tiempo (p ≤ 0.001) y el costo de la inspección inicial (p ≤ 0,001) son mayores que los de la inspección de retorno.

**Conclusión:**

el costo de la etapa de “inspección in loco” subsidiará el Consejo en el proceso de toma de decisiones buscando la eficiencia en la asignación de los recursos humanos requeridos en el proceso de inspección.

## INTRODUCTION

The Regional Nursing Councils (COREN) have the mission of governing over the activities carried out by nursing professionals in the exercise of the profession, in compliance with the legislation of the Federal Council of Nursing (COFEN)/COREN system. The Inspection of Professional Nursing Practice, the main action to achieve this mission, requires the action of human resources, generating costs that need to be identified and efficiently managed^(1–2)^.

The Supervision of Professional Nursing Practice has the educational, preventive, disciplinary and correctional concepts as its target, aiming at the defense of society and the quality of nursing care, and can be exercised through several procedures, including inspection, hearing, meeting, and lectures^(3)^.

Inspection is one of the main procedures and consists of the act planned in advance and assigned to the inspector, who has to go to a health institution and inspect the functioning and organization of the Nursing Service (NS), guiding the Nurse (TN) who is in charge of the technical issues, and the other Nursing professionals present, when feasible, on compliance with legislation related to professional practice and the provision of safe Nursing care for patients and employees, to prevent the occurrence of violations of the laws regulating Nursing practice^(2)^. In addition, when irregularities and illegalities are identified, notifications are applied with a deadline for regularization^(3)^.

The inspector, an agent trained to carry out the inspection in health institutions, is hired through a public examination, shall have a degree in Nursing and have at least a graduate certificate in the field of Nursing or in health services administration, as well as proven professional experience of at least three years. Among their activities there is the performance of inspections in health institutions that include nursing professionals, issuing notifications and notices of infraction, providing specific guidelines for professional practice and issuing technical, ethical, and scientific opinions^(1,3)^.

The inspections are divided into the initial ones and those of return and, according to the normative document of COREN-SP, the initial *in loco* inspection is carried out in an institution that made a new registration in COREN-SP; those with an existing registry, but whose corporate name and National Corporate Taxpayer Register have been changed, or those with an existing registry, but the last inspection having taken place more than a year ago, requiring updating of the activities carried out in the NS and the dimensioning calculation. *In loco* return inspection is conducted when all deadlines notified in the initial inspection are reached, to investigate the resolution of technical, ethical, and administrative inconsistencies notified in the initial inspection, which had a deadline to be resolved, corroborating the rite established by COFEN’s Resolution No. 617/2019^(3)^.

The feasibility of Supervision of Nursing Practice requires the payment of salaries to the inspectors, car rental (for the inspector’s travels), and daily wages (in cases where the inspector needs to go to a health institution located more than 100 km from the subsection of origin); and costs associated with expenses with paper, postage, among others. When seeking information on the costs related to the inspection process, only isolated, sporadic and discontinued records of partial costs with the salaries of inspectors, car rentals, and daily wages were identified, with no systematization of obtainment of such information and corresponding documentation.

In view of COREN’s mission of disciplining and inspecting Nursing Practice supported by ethical and legal requirements^(3)^ and the indispensability of proper cost management, the aim was to identify the average direct cost (ADC) related to direct labor (DL) of the inspectors involved in the “*in loco* inspection” of the inspection process carried out at the Headquarters of COREN-SP.

## METHOD

### Type of Study

Quantitative, exploratory-descriptive research, in the form of a single case study.

### Local

The study was carried out at the Inspection Management (GEFIS) of the Headquarters of COREN-SP, located in the central area of the city of São Paulo. Initially, ADC calculation had been foreseen through observations not participating in the *in loco* inspections for measuring the time spent by inspectors. However, in March 2020, these inspectors’ actions were directed exclusively to specific aspects related to the COVID-19 Pandemic, with all activities related to the inspection process being suspended, with no return forecast.

Therefore, to allow the continuity of this study, it was decided to use the information contained in the computerized system of COREN-SP related to initial and return *in loco* inspections, carried out, as recommended by Cofen Resolution No. 617/2019^(3)^, in typical months.

### Sample

In 2019, the 72 inspectors working at COREN-SP carried out 6,415 inspections^(4)^ corresponding, on average, to seven inspections/per inspector/month. From this historical series, a non-probabilistic convenience sample, consisting of records of initial and return “*in loco* inspections*”* took place from January 13, beginning of actions based on Cofen Resolution No. 617/2019^(3)^, to March 13, 2020, the last day of inspections, before the Covid-19 Pandemic period, which were carried out by 13 inspectors working at the Headquarters of COREN-SP.

### Data Collection

Data collection was carried out with the leaders of the Information Technology Management and the Human Resources Management, who provided information regarding the inspections carried out between January 13 and March 13, 2020 (code and institution name; location [city name]; type of establishment; legal nature [public or private]; type of inspection [initial or return]; name of inspector; date and time of start and end of inspection [converted into minutes]) and the wage bill of inspectors working at the Headquarters of COREN-SP.

### DL Calculation

Direct Labor refers to the personnel working directly to obtain a product or service provided, as long as there is the possibility of identifying the time spent and who performed the work. It consists of salaries, social charges, provisions for vacations, and year-end mandatory bonus^(5)^. DL was obtained by multiplying the time spent by inspectors participating in the “*in loco* inspection*”* step of the inspection process at the unit cost of the DL^(6)^.

The calculation of the weighted average of the monthly wages (144 hours/month) of the inspectors corresponded to BRL 23,167.30/144 from which the average cost per hour (BRL 115.84) and per minute (BRL 1.93) was obtained.

### Ethical Aspects

The study was approved in 2021 by the Research Ethics Committee of Escola de Enfermagem da Universidade de São Paulo with Opinion number 4.655.626, meeting the specifications of Resolution No. 466, of December 12, 2012, of the National Health Council.

## RESULTS

From January 13 to March, 03, 2020, GEFIS’s inspectors carried out 182 initial and 98 return *in loco* inspections, totaling 280 inspections. In the initial ones, the types of settings inspected in greater quantity were Basic Health Units – *UBS* (42.9%), Specific Outpatient Clinics (22.5%), Long Stay Institutions for the Elderly – LSIE (7.7%), Medical Clinics (5.5%), and Emergency Care/Emergency Room – EC/ER (3.8%); and the return ones were at *UBS* (27.6%), Hospitals (20.4%), LSIEs (13.3%), Specific Outpatient Clinics (11.2%) and ED/ER (10.2%).

Most (169 – 60.0%) health institutions inspected were public, with the main ones being: UBS (62.1%), Specific Outpatient Clinics (16%), EC/ER (6.5%), and Psychiatric Units (6.5%). Among the private institutions (111 – 40.0%), LSIE (23.4%), Specific Outpatient Clinics (22.5), Hospitals (16.2%) and Medical Clinics (11.7%).

According to Table 1, the average time taken to carry out initial *in loco* inspection was 171.85 (SD = 72.706) minutes, and for the return *in loco* inspection, 132.72 (SD = 67.82) minutes. The average time of initial *in loco* inspection was 1.29 times higher than the return *in loco* inspection.

**Table 1.** Time distribution in minutes for initial and return *in loco* inspections – São Paulo, SP, Brazil, 2021.

Variable	Type of *in loco* inspection	Position, central tendency and variability measures	
**Time in minutes**	**Initial (N = 182)**	Mean	171.85
		Median	155
		Standard deviation	72.71
		Minimum – Maximum	60 – 555
	**Return (N = 98)**	Mean	132.72
		Median	120
		Standard deviation	67.82
		Minimum – Maximum	40 – 360

In Table 2, it can be observed that ADC in initial *in loco* inspection corresponded to BRL 331.67 (SD = 140.32) and the return *in loco* inspection ADC to BRL 256.16 (SD = 130.90). It is observed that initial *in loco* inspection ADC was 1.29 times higher than in the return *in loco* inspection.

**Table 2.** Distribution of the cost of initial and return *in loco* inspections – São Paulo, SP, Brazil, 2021.

Variable	Type of *in loco* inspection	Position, central tendency and variability measures	
**Cost**	**Initial (N = 182)**	Mean	331.67
		Median	299.15
		Standard deviation	140.32
		Minimum – Maximum	115.80 – 1071.15
	**Return (N = 98)**	Mean	256.16
		Median	231.6
		Standard deviation	130.9
		Minimum – Maximum	77.20 – 694.8

When the variables “time” and “cost” of initial and return *in loco* inspections are analyzed, it can be observed in Table 3, using a confidence interval of 95%, with application of the non-parametric test *Wilcoxon-Mann-Whitney,* that the level of alpha significance was 0.05, considered statistically significant, that is, the time (p ≤ 0.001) and the cost of initial *in loco* inspection (p ≤ 0.001) are greater than in return *in loco* inspection.

**Table 3.** Distribution of time and cost of initial and return *in loco* inspections inspections – São Paulo, SP, Brazil, 2021. (N = 280)

Variables		Total of inspections (N = 1)	Initial *in loco* inspection N = 182 (65%)	Return *in loco* inspection N = 98 (35%)	p-value
**Time in minutes**	Mean ± SD	158.2 ± 73.3	171.9 ± 72.5	132.7 ± 67.8	≤0.001
	Median	142.5	155	120	
	Min – Max	40 – 555	60 – 555	40 – 360	
**Cost in *Reais***	Mean ± SD	305.24 ± 141.54	331.67 ± 140.32	256.16 ± 130.90	≤0.001
	Median	275.03	299.15	231.6	
	Min – Max	77.20 – 1071.15	115.80 – 1071.15	77.20 – 694.80	

Mann-Whitney U Test.

## DISCUSSION

During the study period, 280 NS inserted in several health institutions were inspected, which were included in the Annual Inspection Plan, aiming to meet the guidelines of the COFEN Multi-Annual Plan, considering the allocation of budgetary resources from COREN-SP. The classification of health facilities is based on the Department of Informatics of the Unified Health System (DATASUS) guidelines^(7)^, which considers the type of establishment according to its purpose:

– Specific Clinic: Institutions dedicated to specific outpatient care (only one specialty or care area), such as: infectious diseases, ophthalmology, rehabilitation;
– Diagnoses Center: Institutions that perform diagnostic tests or complement the treatment, such as tomography, magnetic resonance, among others;
– Medical Clinics: Institutions dedicated to the clinical treatment of different specialties;
– Cooperative: Company that offers cooperative professionals to provide care in healthcare facilities;
– Hospital: Institution that provides care in several medical specialties, as well as specialized services, such as hemodialysis service, chemotherapy, laboratory, among others;
– EC/ER: Unit dedicated to the care of cases, with or without risk of death, whose health problems need immediate assistance; and
– *UBS*/Family Health Strategy/Health Center: Institutions dedicated to providing basic and comprehensive care to a specific population and, in some cases, offering therapeutic diagnostic support services and 24-hour EC.

In view of this diverse scenario, the inspector needs to know and recognize the main activities carried out in the NS, according to the type of setting, and check whether they are in compliance with the legal precepts of the COFEN/COREN system, in addition to complying with the specific legislation for each service, as examples, the LSIE^(8)^ and dialysis services^(9)^, directing the relevant guidelines to the type of complexity of care provided by the nursing team, which is directly associated with the calculation of staff dimensioning.

In this study, regarding the legal nature of health establishments, it was evidenced that 60% of the NSs were inserted in public institutions and 40% in private institutions. It should be noted that, in COREN-SP, there are more than 20,000 registered health institutions, 46% of which are public and 54% private, with the following distinctions being made: Hospitals, *UBS*, EC, and other types of public institutions (federal, state and municipal); philanthropic and private, based on the classification suggested to CORENs through COFEN Resolution No. 617/2019^(3)^.

In the professional practice of one of the authors of this article, it was possible to observe that the management of state, municipal hospitals, and of *UBSs*, of public administration or public-private partnership (hybrid model), managed by Social Health Organizations (*OSS*), undergoes different impacts in organizational results and, consecutively, in the NS, mainly in decision-making, structuring of work processes and forecast and provision of the nursing staff, also impacting the time spent by the inspector in the *in loco* inspection, by finding different realities, with inconsistencies and weaknesses, which generate more analysis and guidance regarding the irregularities identified.

On the one hand, institutions with Public-Private Partnerships, with *OSS*, demonstrate feasibility and speed regarding public service processes, in what regards hiring of human resources, reorganization and purchase of material resources, and adequacy of physical resources, enhancing assertive development of nursing work. On the other hand, institutions of public administration have, for the most part, *deficit* of nursing staff, who are hired only through the performance of tests, so that, often, the insufficiency of staff is not resolved as soon as the situation requires; scrapped materials and equipment; neglected physical resources; failures in the planning and evaluation of services, compromising the efficiency and effectiveness of the service and, consequently, NSs’ managerial and care quality.

This scenario makes the inspector add other actions to the activities carried out in the initial and return *in loco* inspections, such as: training directed at TN on the elaboration of the calculation of personnel dimensioning, organization and management of documents and control of processes; team training on nursing records, and meeting with managers, aiming to present the irregularities found and agree deadlines for regularization.

According to a study carried out with seven *OSS* managers, the main potentials of this model are: speed in solving problems related to human resources (including nursing professionals) and purchasing, use of the budget assertively, reducing costs, and definition, organization, and monitoring of work processes, in accordance with the established goals^(10)^. This result is ratified with the agreement of contracts, considering the hybrid management and covering 23 geographic areas in the State of São Paulo^(11)^.

Also, in line with previous studies, a study carried out to analyze the performance of the management carried out by the Public Administration, versus *OSS*, in the State of São Paulo, from 2013 to 2016, showed that the institutions managed by the latter model had superior results relative to the average length of hospital stay, occupancy rate, cesarean rate, hospital infection and production costs^(12)^.

Narrative literature review, when analyzing 33 publications, indicated results that were divergent from those previously presented. It concluded that there is no consensus among the few comparative studies, as public hospitals managed by OSS, when compared to hospitals managed by the Government, in the State of São Paulo, showed greater economic efficiency and better management of human resources. On the contrary, public services in Curitiba (Paraná) showed similar performance under the management of the Public Administration^(13)^.

To carry out the 182 initial *in loco* inspections, the inspectors used a guide, contained in the Term of Inspection (*TF)* ^(3)^, in which the actions, findings, notifications and recommendations of the action were recorded. The main points observed/analyzed in the actions were: inspection in all sectors where Nursing is active; characterization of the institution and the NS (hours, number of categories, distribution of professionals and activities performed); compliance with legislation, highlighting aspects related to the Nursing Process, the Code of Ethics of Nursing Professionals, and article 10 of Resolution 509/2016^(14)^; guidance on identified irregularities and/or illegalities, notification with a deadline for regularization and the proposition of educational actions, for example, guiding the nurse on the step-by-step on how to prepare the calculation of nursing staff dimensioning.

For a similar purpose, the Medical, Physiotherapy, and Occupational Therapy councils carry out inspections, aimed at identifying whether the professional practice is being performed in accordance with current legislation, notifying irregularities, establishing deadlines for adjustments, in addition to developing educational actions^(15–16)^.

When sanitary, structural, and labor irregularities are detected that cause damage to the Nursing care provided and for which COREN-SP has no prerogative for resolution, the inspector registers them in the *TF* and, subsequently, they are forwarded by COREN to the competent authorities for investigation, such as the Health Surveillance and the State Prosecutor’s Office^(3)^.

It should be noted that the guide from COREN-SP used by GEFIS is the same for all types of NS, as the main focus is to check the compliance with the legislation of the COFEN/COREN-SP System, based on the analysis of the activities carried out. In the same direction, a study carried out on the specificities of supervision of the Social Worker’s professional practice demonstrates the use of a single guide to collect data on the operating procedures and working conditions of these professionals^(17)^.

In this respect, the National Health Surveillance Agency (ANVISA) differs from the COFEN/COREN-SP System and the Federal Council of Social Services, as it uses specific guides for certain institutions/services. In 2020, it launched eight documents including a guide and a risk assessment form designed to analyze the activities carried out in the operating room, material and sterilization center, dialysis and intensive care unit, aiming to standardize the inspection processes in health services^(18)^. The Federal Council of Medicine is similar to the Brazilian Health Regulatory Agency (ANVISA), as it also uses different guides in inspections carried out in hospitals, ERs, and other types of health settings^(15)^.

It is understood that the use of a single guide for all types of health settings has the advantage of speed in the development of initial or return *in loco* inspection of the NSs, as the inspector must comply with the legal questions contained in the *TF* and, as a disadvantage, the failure to make a more in-depth analysis of a more specific questioning of a given NS, due to the fact that the inspector has no prior experience or peculiar knowledge in that area, incurring in putative impairment to the assistance.

Ninety-eight return *in loco* inspections were carried out, after the end of all notified deadlines, to investigate the resolution of technical, ethical, and administrative inconsistencies found and notified in the initial *in loco* inspection, which had a deadline to be solved, corroborating the rite established by Cofen Resolution No. 617/2019^(3)^. If the inspectors found new irregular and/or illegal situations, related to Nursing practice, during the return *in loco* inspection, they could notify them, establishing new deadlines for its resolution.

In this study, it was found that the average time taken to carry out the initial *in loco* inspection was longer (171.85 minutes) than the return *in loco* inspection (132.72 minutes). This difference can be explained by the fact that, to carry out the initial *in loco* inspection, the inspector needs to request information and evaluate all the questions contained in the *TF*, as well as inspect the sectors in which Nursing operates. On return *in loco* inspection, the inspector’s main target is to check if the inadequacies notified in the initial *in loco* inspection were solved and fill in the *TF*, regarding specific issues.

Other elements are highlighted that can contribute to the initial *in loco* inspection time being greater than the return *in loco* inspection, particularly the size of the institution; complexity of services provided and, consecutively, of activities performed by nursing workers; number of nursing professionals, specific knowledge and previous experience in the training area of each inspector.

Research carried out to raise the ADC of activities performed by nursing professionals regarding the documentation of the Nursing Process, in a Medical Clinic Unit of a university hospital, resulted in an average time spent by nurses of 39.40 (SD = 14.61) minutes in activities related to admission (interview, physical examination, printing of reports), and 19.13 (SD = 8.36) minutes for activities related to the follow-up of hospitalized patients (organization of the documentation of the Nursing History and notes taken)^(19)^.

Brazilian studies^(19,20,21,22,23)^ which sought to identify the ADC of procedures performed by nursing professionals in care units, such as the intensive care unit (severely burned patients and patients with acute kidney injury), material and sterilization center (reprocessing of surgical drapes), and medical and surgical clinic (patients with incidence for the development of pressure injuries), observed that the duration of procedures performed by nurses, technicians and nursing assistants were similar in terms of variability, due to the complexity of the procedures and the professional’s knowledge and dexterity.

An original study carried out in 27 Family Health Units, covering 10 states in Brazil, determined the average time standards for interventions/activities carried out by nursing professionals. As for the activities performed by the nurse, the average time varied between 75.0 (administrative meeting) and 63.0 minutes (evaluation of professional care) and 13.0 (development of process/administrative routines) and 11.0 minutes (organization of the work process)^(24)^.

As expected, ADC related to the DL of the inspectors to enable the initial *in loco* inspection was higher than the return *in loco* inspection ADC. Despite recurrent searches of the national and international literature, no similar studies were found that would allow the discussion of the specific financial- economic results obtained, indicating the knowledge gap that needs to be increased and verticalized based on research developed by other CORENs, including activities performed before and after *in loco* inspection. However, there is evidence of research that funded the ADC of nursing professionals involved in carrying out processes and procedures^(25,26,27,28)^.

A study carried out to identify the total ADC of procedures performed by nursing professionals to severely burned patients in an intensive care unit indicated a variation in the duration of the “curative” procedure between seven and 264 minutes, with an average of 72.52 (SD = 54.37) minutes, and obtained the ADC with DL of nurses corresponding to US$ 26.00 (SD = 24.90), ranging from US$1.75 to US$115.50^(20)^.

When analyzing the time and cost of initial and return *in loco* inspections variables, statistical significance was observed, namely, the time (p ≤ 0.001) and the cost of initial *in loco* inspection (p ≤ 0.001) are greater than in return *in loco* inspection. It is indicated that the present study, original in Brazil, when highlighting the economic and financial aspects related to the DL of the inspectors involved in carrying out the initial and return *in loco* inspections, contributes to the COFEN/CORENs System by generating knowledge that can support decision- making to increase efficiency and effectiveness of human resources required in the inspection process.

It should be noted that a study carried out to identify the direct cost of maintaining the permeability of central venous catheters showed that the lack of knowledge of the costs of processes carried out in organizations hinders the decision- making process on where and how to properly allocate the resources required for the provision of health services^(28)^.

Therefore, it is important for the manager to map the organizational processes to identify the activities that add and those that do not add value, in addition to mastering the costing principles and methods and their functionality, and thus manage resources efficiently and effectively by means of assertive decision-making related to targeting their strengths and possible corrections of distortions and waste^(29)^.

Finally, as a contribution to the COFEN/COREN System, this study highlighted the economic and financial aspects related to the DL of the inspectors required to carry out the initial and return *in loco* inspections. This kind of research can help COREN’s managers in the preparation of the annual planning of inspections, considering the budget forecast, the establishment of goals, the dimensioning of inspectors required, and the implementation of operational and strategic indicators. In addition, they allow the projection of opportunities for rational allocation of the inspector’s specialized ADC, in favor of finalist activities, increasing the scope of *in loco* inspections, covering a greater number of Nursing professionals and increasing the performance of educational actions against the irregularities found.

## CONCLUSION

Initial *in loco* inspection ADC was BRL 331.67 (SD = 140.32), ranging from BRL 115.80 and BRL 1071.15 and median of BRL 299.15, and the return *in loco* inspection was R$256.16 (SD = 130.90), ranging from R$77.20 to R$694.80, with a median of R$231.60.

Time and cost variables analysis of initial and return *in loco* inspections showed an alpha significance level of 0.05, and it was possible to statistically state that the time (p ≤ 0.001) and the cost of initial *in loco* inspection (p ≤ 0.001) are higher than those for return *in loco* inspection.
