# ERRATUM: *In loco* nursing practice inspection costs in a Brazilian setting

**DOI:** 10.1590/1980-220X-REEUSP-2021-0382er

**Published:** 2022-04-11

In the article “*In loco* nursing practice inspection costs in a Brazilian setting”, with DOI code number https://doi.org/10.1590/1980-220X-REEUSP-2021-0382, published at Revista da Escola de Enfermagem da USP [online], v.56: e20210382, on the page 3:

**Where it was written:**

“The calculation of the weighted average of the monthly wages (144 hours/month) of the inspectors corresponded to BRL 23,167.30/144 from which the average cost per hour (BRL 115.84) and per minute (BRL 1.93) was obtained.”

**Should read:**

“The calculation of the weighted average of the monthly wages (200 hours/month) of the inspectors corresponded to BRL 23,167.30/200 from which the average cost per hour (BRL 115.84) and per minute (BRL 1.93) was obtained.”

